# Presence and Persistence of *Salmonella* in Water: The Impact on Microbial Quality of Water and Food Safety

**DOI:** 10.3389/fpubh.2018.00159

**Published:** 2018-05-30

**Authors:** Huanli Liu, Chris A. Whitehouse, Baoguang Li

**Affiliations:** ^1^Branch of Microbiology, Arkansas Laboratory, Office of Regulatory Affairs, United States Food and Drug Administration, Jefferson, AR, United States; ^2^Division of Molecular Biology, Center for Food Safety and Applied Nutrition, United States Food and Drug Administration, Laurel, MD, United States

**Keywords:** irrigation water, agricultural water, *Salmonella*, produce safety, prevalence, pathogen detection, foodborne outbreaks

## Abstract

*Salmonella* ranks high among the pathogens causing foodborne disease outbreaks. According to the Centers for Disease Control and Prevention, *Salmonella* contributed to about 53.4% of all foodborne disease outbreaks from 2006 to 2017, and approximately 32.7% of these foodborne *Salmonella* outbreaks were associated with consumption of produce. Trace-back investigations have suggested that irrigation water may be a source of *Salmonella* contamination of produce and a vehicle for transmission. Presence and persistence of *Salmonella* have been reported in surface waters such as rivers, lakes, and ponds, while ground water in general offers better microbial quality for irrigation. To date, culture methods are still the gold standard for detection, isolation and identification of *Salmonella* in foods and water. In addition to culture, other methods for the detection of *Salmonella* in water include most probable number, immunoassay, and PCR. The U.S. Food and Drug Administration (FDA) issued the Produce Safety Rule (PSR) in January 2013 based on the Food Safety Modernization Act (FSMA), which calls for more efforts toward enhancing and improving approaches for the prevention of foodborne outbreaks. In the PSR, agricultural water is defined as water used for in a way that is intended to, or likely to, contact covered produce, such as spray, wash, or irrigation. In summary, *Salmonella* is frequently present in surface water, an important source of water for irrigation. An increasing evidence indicates irrigation water as a source (or a vehicle) for transmission of *Salmonella*. This pathogen can survive in aquatic environments by a number of mechanisms, including entry into the viable but nonculturable (VBNC) state and/or residing within free-living protozoa. As such, assurance of microbial quality of irrigation water is critical to curtail the produce-related foodborne outbreaks and thus enhance the food safety. In this review, we will discuss the presence and persistence of *Salmonella* in water and the mechanisms *Salmonella* uses to persist in the aquatic environment, particularly irrigation water, to better understand the impact on the microbial quality of water and food safety due to the presence of *Salmonella* in the water environment.

## Introduction

*Salmonella* is a natural inhabitant in the gastrointestinal tract of many animals, including birds, reptiles, livestock, and humans (1–7). Salmonellosis caused by nontyphoidal *Salmonella* ranks among the highest in all gastroenteritis cases linked to food consumption, affecting the health of approximately one million people annually in the United States alone (8, 9), resulting in medical costs of $3.7 billion. It is estimated that *Salmonella* species causes 93.8 million cases of gastroenteritis worldwide annually with 155,000 deaths (5−95th percentile, 39,000–303,000) (10). The causative source for salmonellosis has traditionally been attributed to animal origin (11, 12), including meat, eggs, and other poultry products, which has attracted considerable regulatory attention and enormous mitigation efforts (2, 9). In recent years, the number of foodborne outbreaks due to nontraditional sources of the pathogen such as domestic or imported fresh fruits, vegetables, spices, and nuts has been increasing (9, 13–15). A recent report from the Centers for Disease Control and Prevention (CDC) suggested nearly half (46%) of foodborne illnesses and 23% of deaths were associated with produce consumption (16). For instance, during July 2015 to February 2016, a *Salmonella* outbreak associated with consumption of imported Mexican cucumbers caused illness in at least 907 people, with six deaths in 40 states within the United States (17).

Consumption of more fresh fruits and vegetables has been advocated as a healthier diet habit because raw, or less processed, fruits and vegetables are good sources of vitamins, fiber, and other beneficial nutrients (18, 19). Since the early 1970s, the demand for fresh produce in the US has been on the rise continuously, and the estimated increase of per capita consumption of fresh fruits and vegetables from 1982 to 1997 reached approximately 32% (13). The consumption of vegetables grew more rapidly than fruits from 1976 to 2009 (20). A recent investigation by the US Department of Agriculture also suggested Americans consumed more fresh produce in 2015 than in 1970 (21) As a result, it is expected that more produce-related outbreaks of disease will occur even if the contamination sources and rates stay at the present levels. This has attracted the attention of the legislative branch and food safety regulatory agencies in the United States. The Food Safety Modernization Act (FSMA), which was enacted in 2011, emphasizes the significance of produce safety. In response to that, the US Food and Drug Administration (FDA) issued the Produce Safety Rule (PSR) in January 2013 to establish science-based standards for the growing, harvesting, packing, and holding by domestic and foreign farms of produce consumed in the United States (22).

The causative pathogens for produce contaminations include viruses, bacteria, and parasites. Contamination may occur at various stages during the production process (2). *Salmonella* has been regarded as the primary pathogen for causing produce-related foodborne outbreaks. Pathogenic bacteria carried by produce that can lead to an outbreak may involve multiple external sources and production stages. For instance, at the pre-harvest stage, *Salmonella* can come from specific agricultural practices such as using animal manure as fertilizer (1) and others may include using contaminated water for irrigation, pesticide spraying, or anti-frost spraying (2, 23, 24).

Water has been shown as a source of microbial contamination of fresh produce and a vehicle for pathogen transmission (23, 25). FSMA defines “agricultural water” partially as water that is “intended to, or is likely to contact covered produce or food contact surfaces” (22). Irrigation water consists of a major component of agricultural water. The sources of irrigation water can come from ground water, surface water, or municipal water. Irrigation water can be applied to produce through various ways including drip tape, furrows, and overhead sprinklers (26). In this review, we will primarily focus on the presence, survival, persistence, and source of *Salmonella* in surface water, particularly irrigation water, to help us better understand the impact on the microbial quality of water and food safety due to the presence of *Salmonella* in the water environment.

## Produce as a vehicle for transmission of *Salmonella*

**Figure 1:**
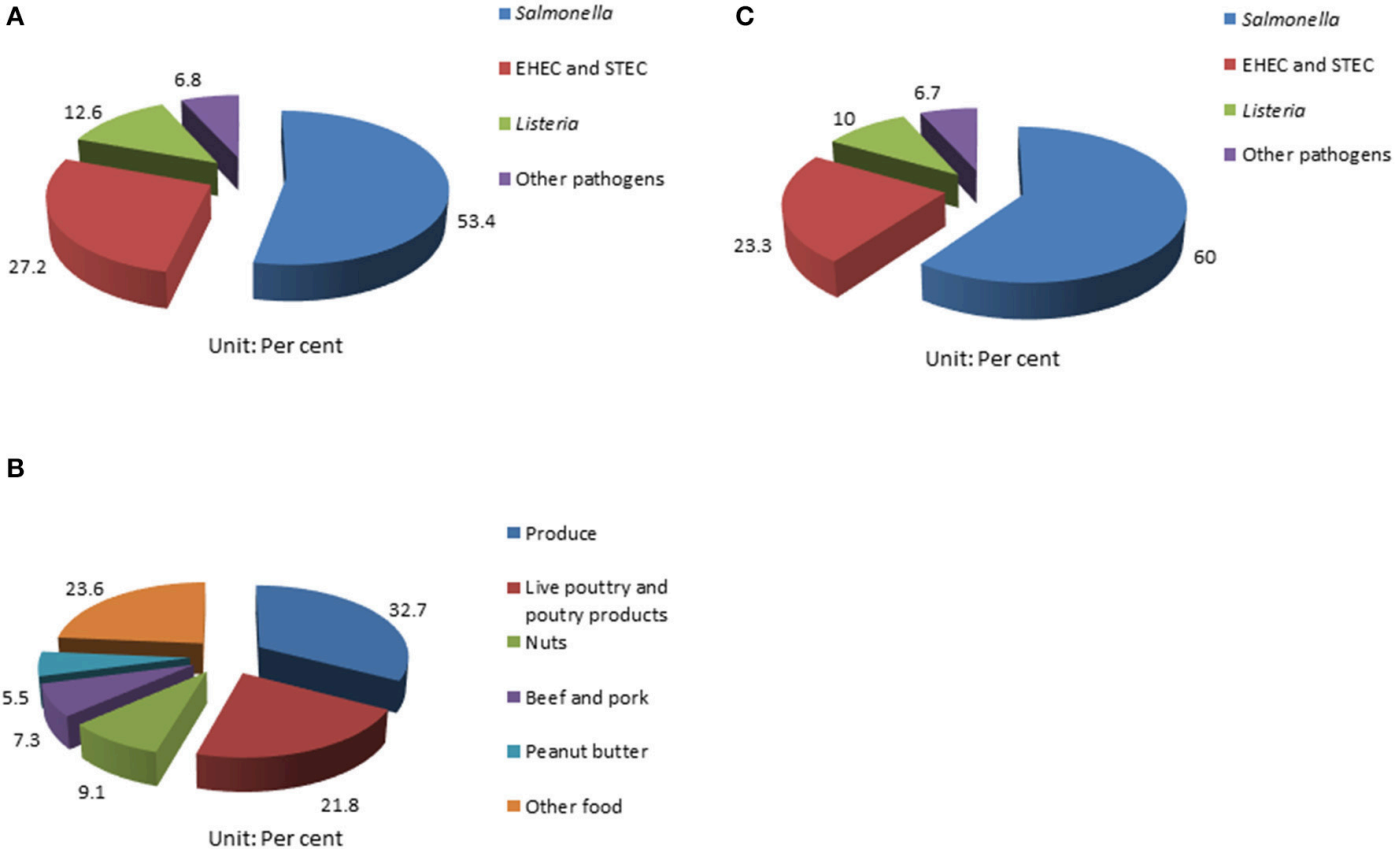
Summary of surveillance of foodborne pathogen outbreaks from CDC (29). **(A)** Multistate foodborne outbreaks caused by *Salmonella* rank highest in all the pathogens surveilled; **(B)** the percentage of multistate *Salmonella* outbreaks linked to produce from 2006 to 2017; and **(C)** and *Salmonella* contributes to 60% of all produce-related outbreaks caused by different pathogens surveilled.

Foodborne outbreaks associated with fresh produce in the United States have been on the rise in the last few decades; and *Salmonella* has been recognized as the primary causative pathogen (1, 11, 13, 27). According to a report from the CDC, 31 *Salmonella* outbreaks from 2002 to 2003 were associated with fresh produce; while 29 were poultry related (2, 28). Also, *Salmonella* contributes to 53.4% (55/103) of the foodborne disease outbreaks documented by CDC among all pathogens investigated from 2006 to 2017 (Figure 1A); 32.7% (18/55) of the multistate foodborne *Salmonella* outbreaks were associated with produce (Figure 1B); and 60% (18/60) of the fresh produce-related outbreaks were attributed to *Salmonella* among all the pathogens involved (Figure 1C) (29). Many common types of produce have been implicated in *Salmonella*-related outbreaks, including beans, alfalfa sprouts, tomatoes, hot peppers, lettuce, cucumbers, cantaloupes, water melons, papayas, and mangoes (1, 2, 29–32).

*Salmonella* is originally from, and has adapted to, the microenvironment of the gastrointestinal tract of animals. Once released from the animal gastrointestinal tract or other sources with feces or exudates, *Salmonella* can be carried to surface waters through rainfall and surface runoffs, survive many challenges such as ultraviolet (UV) radiation from sunlight, poor nutrients, the changes in pH, and temperature (33–35). After attaching to produce, *Salmonella* must persist under these adverse environmental conditions at a sufficient concentration to cause human illness (13). The surface of various fruits and vegetables provides a niche for numerous bacterial species, such as epiphytes, plant pathogens, and opportunistic human pathogens, such as *Salmonella* (36). Interestingly, some plant pathogenic bacteria, such as *Pectobacterium carotovorum*, can promote the growth of *Salmonella* by macerating plant tissue and providing nutrients (9, 37), whereas other bacterial species such as *Panebacillus* spp. on tomatoes and cilantro may inhibit or even kill *Salmonella* (38, 39). Thus *Salmonella* has to survive the inhibition from these microbes and outcompete them for the acquisition of nutrients and space (33).

The mechanisms for how *Salmonella* are carried in irrigation water or from other sources interact with plants, survive or persist on these intermediate hosts have become an interesting topic in recent years. Studies indicate *Salmonella* can be internalized into tomato plants through different routes and may possibly colonize and employ the plant as an alternative host (2, 9, 36). The formation of biofilm-like structures on the surface of roots, the colonizing regions of emerging lateral roots and wounded tissues has been reported (40, 41). Moreover, *Salmonella* inoculated into soil or blossoms can be recovered from the fruit at low internal levels, suggesting its ability to colonize and internalize tomato plants, and however, survival ability with different serovars varies in the soil and in different parts of the plants (42). *Salmonella* was also recovered from tomato fruits after it was introduced into the plant by stem injection or by flower inoculation (43). Stine et al. demonstrated that *Salmonella* was still detectable for at least 14 days after inoculation, indicating the bacteria probably developed a mechanism to adapt and survive within this hostile environment (44).

Recent studies have further suggested *Salmonella* may not only passively use plants as a “shelter” for survival, but a growing body of evidence has indicated it also may have evolved mechanisms for active adhesion and escape of plant immunity systems and actively internalize and proliferate in some plants, such as the tomato (9, 42, 45–47). It was found that *Salmonella* populations inoculated onto the exterior of tomatoes can increase in numbers at suitable humidity and temperature and such bacterial growth is serovar dependent (48). This may explain why some serovars, such as Newport, Montevideo, and Saintpaul, are more frequently linked to *Salmonella* outbreaks (2). A latest study suggested that the mechanisms *Salmonella* requires to colonize tomato plants are similar to phytopathogens, such as biosynthesis of amino acids, lipopolysaccharides (LPS), and nucleotides, indicating the flexibility of this pathogen to fit different hosts (49). The studies on *Salmonella* entry into inner leaves indicated that while trichomes are postulated as preferential colonization sites (50, 51), stomata are shown to be entry points *Salmonella* utilize for penetration of lettuce leaves (52, 53). The interaction of *Salmonella* with *Arabdopsis*, potato, sprouts and other plants are also reported and reviewed by Schikora et al. (47).

Preservation of taste, nutrients, and other desirable qualities of produce demand minimum processing, including avoidance of heating and sanitation with disinfectant to leafy vegetables and sprouts (2, 54), if washing is inadequate, the contamination acquired in field or production stage and postharvest easily transit from field to the table. Washing with chlorinated water, water-dip disinfection procedures may be applied to fruits such as melons, mango, and papaya. But if the disinfection efficiency of chlorinated water is not adequately monitored, the contaminated *Salmonella* can still be attached; and the rough surfaces for some types of melon like Cantaloupes can increase the adherence of bacteria and compromise the effect of washing (15, 30, 31, 55). Nowadays, the precut ready-to-eat vegetables and fruits are widely available to consumers in grocery stores; and poor hygiene and sanitary practice in preparation of these foods can bring additional chances for bacterial contamination (15, 56, 57). Therefore, the elimination of *Salmonella* contamination of fresh produce from fields to forks is almost impossible because of the above-mentioned loopholes. In general, the frequent occurrence of *Salmonella* outbreaks associated with produce and the scarcity of approaches available for sanitizing contaminated produce underscore the urgency for development of more efficient preventive control measures that can be applied at an earlier stage in the produce production.

## Irrigation water as a source or a vehicle for transmission of *Salmonella*

The original source of *Salmonella* on produce may come from soil, manure, irrigation water, and contact with reptiles, birds, or other small animals (23, 24, 58). Irrigation water has drawn considerable attention in recent years, and studies have implicated irrigation water as a source of *Salmonella* contamination (23, 24, 59). This hypothesis has been previously reviewed (5, 23, 24, 27, 60, 61) and appears to be supported by trace-back investigations of produce-related *Salmonella* outbreaks (62, 63). The 2005 multistate *Salmonella* outbreak involving tomatoes was caused by Newport serotype, and a trace-back investigation linked this strain to an outbreak associated with tomatoes that occurred 3 years earlier. Furthermore, the isolates from these two outbreaks were shown to be genotypically identical by pulsed-field gel electrophoresis (PFGE), and the same bacterial strains were found present in a pond the farm used for irrigation (62). A case control study of a 2008 multistate outbreak identified *Salmonella* Saintpaul on serrano and jalapeño peppers and was also present in irrigation ponds (63). Another study indicated that the PFGE patterns of some of the isolates from irrigation ponds of produce farms in southern Georgia were indistinguishable from strains that were associated with the *Salmonella* Thompson outbreaks in 2010, 2012, and 2013, *Salmonella* Enteritidis outbreaks in 2011 and 2013, and a *Salmonella* Javiana outbreak in 2012 (61). The investigation of papaya *Salmonella* outbreak happened from 2006 to 2007 in Australia also found that the river water used for washing papayas was contaminated with *Salmonella* (64).

## Prevalence and source of *Salmonella* in irrigation water

The fresh produce consumption over the last 40 years and number of foodborne disease outbreaks associated with fresh produce has been increasing (15, 18). Irrigation is a critical factor for the production of fruits and vegetables, *Salmonella* present in irrigation waters has been regarded as one of the major source for fresh produce contamination (2, 23, 24), and this has become a public health concern and drawn more attention of food safety regulatory agencies. Irrigation is a critical factor for the production of fruits and vegetables. As identified above, the source of irrigation water can include groundwater from wells, surface water (rivers and irrigation ponds), and treated wastewater (24, 27, 65, 66). Groundwater in wells is naturally filtered by soil and generally has a higher microbial quality (i.e., less microbes present). But it may be compromised by inferior construction or insufficient depth of the well, and may be contaminated from nearby latrines, septic tanks leaching fields, land application of waste water, and rainfalls (27, 67). Incidences of *Salmonella* contamination of ground water is mainly a concern of developing countries, especially in the rural areas, due to poor hygienic conditions, deficiently-structured water supply systems, and inadequate disinfection treatment (68), but occasionally occurs in developed countries as well (69, 70).

Surface waters, which include ponds, lakes, rivers, and streams, account for nearly half of the water used for irrigation in the United States (26). They are more exposed to environmental events such as discharge of sewage, rainfall, animal husbandry, and wildlife, and thus are more susceptible to contamination as compared to groundwater (23, 67). Rivers have been widely used as an irrigation source for agricultural practice (71, 72); river water, however, has been shown to be one of the largest reservoirs of viable *Salmonella* (2, 73). An estimation of *Salmonella* loads from a coastal Mediterranean river of a 16-month period (74) indicated *Salmonella* occurrence up to 95% during high waterflow (21% of the year) such as storm events. The subsequent study discovered *S*. Typhimurium accounted for 33.1% of all isolates recovered from the river in that period (73). In another study (12), *Salmonella* were detected from 57 of 72 (79.2%) water samples monthly collected from six stations of Little River in upper Suwannee Bain of Southern Georgia State of the United States. The recovery of *Salmonella* from rivers exhibited seasonality pattern, with summer time being highest, which is similar to the prevalence of *Salmonella* in irrigation ponds (6, 61). This increased prevalence of *Salmonella* during summer time may be related to multiple environmental factors, such as host shedding, enhanced persistence of *Salmonella* in warm temperature, and increase of storm events (12). In the Suwannee River watershed of southern Georgia, open surface pond waters are the main source of irrigation water. The case rates in this region have been observed to be 1.5-fold higher than the national average (6, 12). Presence of *Salmonella* in irrigation ponds within this region has been surveyed by Li et al. and showed that *Salmonella* was recovered from 50 of 170 (29.4%) water samples collected monthly over 27 months from 10 selected irrigation ponds that serve as water sources for irrigation of vegetables (61). Furthermore, more than half of the isolates were identified as *S*. Newport and antimicrobial susceptibility testing confirmed 16 *S*. Newport isolates were multidrug resistant (MDR), exhibiting resistance to ampicillin, chloramphenicol, streptomycin, sulfamethoxazole, and tetracycline (ACSSuT) and to the 1st, 2nd, and 3rd generations of cephalosporins (cephalothin, amoxicillin-clavulanic acid ceftriaxone) (61). Another monthly survey by Luo et al. on 10 ponds in the same area isolated *Salmonella* from 28.2% of all samples (*n* = 635) and the most common serotypes were Hadar, Montevideo, and Newport. In addition, 98.9% of the strains were reported to be resistant to streptomycin and about 20% were MDR strains (6). The contamination of fresh produce by these nontyphi *Salmonella* species present in irrigation waters could generate detrimental clinical and public health consequence because the increasing antibiotic resistance limits options of treatment after microbial diagnosis. In foodborne disease outbreak settings, the infection of MDR strains may bring excess of mortality and morbidity (75, 76). Moreover, the encodings genes of MDR may be horizontally transferred to other pathogenic bacteria resulting the spreading of antibiotic resistance (75).

The microbial quality of treated municipal water depends on the efficiency of treatments to remove pathogenic enteric bacteria, viruses, and parasites. Prevalence of *Salmonella* in treated effluent has been reported (77). In the United States, only a limited scale of treated municipal water is used for irrigation of crops because of the concern of potential bacterial contamination (24). However, in countries or regions with a shortage of fresh water, wastewater treated to a suitable level can be used as a substitute for ground water or surface water for irrigation purposes, and the microbial quality guidelines adopted for treated wastewater must be the strictest (23).

It is generally accepted that *Salmonella* present in water can be traced back to its animal origins. This pathogen may directly be transported from feces or exudates of wild animals by rain water runoff to rivers or ponds used for irrigation (12). Manure of domesticated animals has long been used to fertilize soil because it is economical and beneficial to the environment. However, studies have indicated that *Salmonella* in manure can survive as long as 231 days and may eventually contaminate produce by rain water splashing and/or by surface irrigation water (78). Sewage effluents contain waste water from human toilets with a high concentration of bacteria and other pathogens (79). The average concentration of *Salmonella* can reach as high as 2.7 × 10^2^ CFU/100 ml (80, 81), which could become a major source of contamination if discharged directly or with inadequate treatment. *Salmonella* and other bacteria in sewage water can be effectively reduced to very low levels with modern treatment methods, but it is not practical to eliminate all the bacteria. When discharged, this will pose another contamination source of *Salmonella* to surface waters (79, 82) as illustrated in the proposed model of *Salmonella* transmission in the environment (5).

## *Salmonella* survival and persistence in irrigation water

The gastrointestinal tract of vertebrates is generally regarded as the natural habitat for *Salmonella* (83), and bacteria released from feces could be transported into aquatic systems by sewage discharges, rainfall events or associated surface runoffs (84). *Salmonella* may survive in an environment with a broad range of pH (4.05–9.5) and can multiply in a broad range of temperatures (7–48°C) (34). In a closed environment at room temperature (25°C), it has been shown that *Salmonella* can survive for up to 5 years in sterile water or a phosphate buffered solution (85). However, the eco-environment of irrigation water, such as river or pond water, is harsher and more complicated and dynamic. Survival and persistence of *Salmonella* in water depends on multiple environmental factors, such as temperature, pH, salt, dissolved oxygen concentration, nutrient availability, interaction with other microorganisms, and exposure to UV light radiation (23, 27, 86, 87). As a result, *Salmonella* viability will decrease in water over time and could survive generally less than 30 days (23). However, many studies now show that biofilms can facilitate the survival of *Salmonella* in water and invertebrates such as free-living protozoa and various vertebrate animals can serve as reservoirs for *Salmonella* and other pathogenic bacteria. For example, Gaertner et al. detected *Salmonella* from water biofilms and crayfish samples from the headwater spring of Spring Lake, Texas and found that *Salmonella* isolates from biofilms collected 23 days apart shared the same Rep-PCR profile, suggesting *Salmonella* infrequently washed into an aquatic system might take up water biofilms as dwellings for long term persistence (88). Sha et al. further demonstrated *Salmonella* isolated from biofilms on a concrete surface exhibited significant microheterogeneity, but remained pathogenic (89). Additionally, *Salmonella* were recovered from sediments in water, and the concentration was higher than in the overlying water. Similar phenomena have also been observed in survival of other bacteria in water. This might be caused by sedimentation and absorption (90–92), which may help explain why continuous prevalence of *Salmonella* over a long period of time was found in river and surface ponds in multiple studies (5, 12).

**Figure 2:**
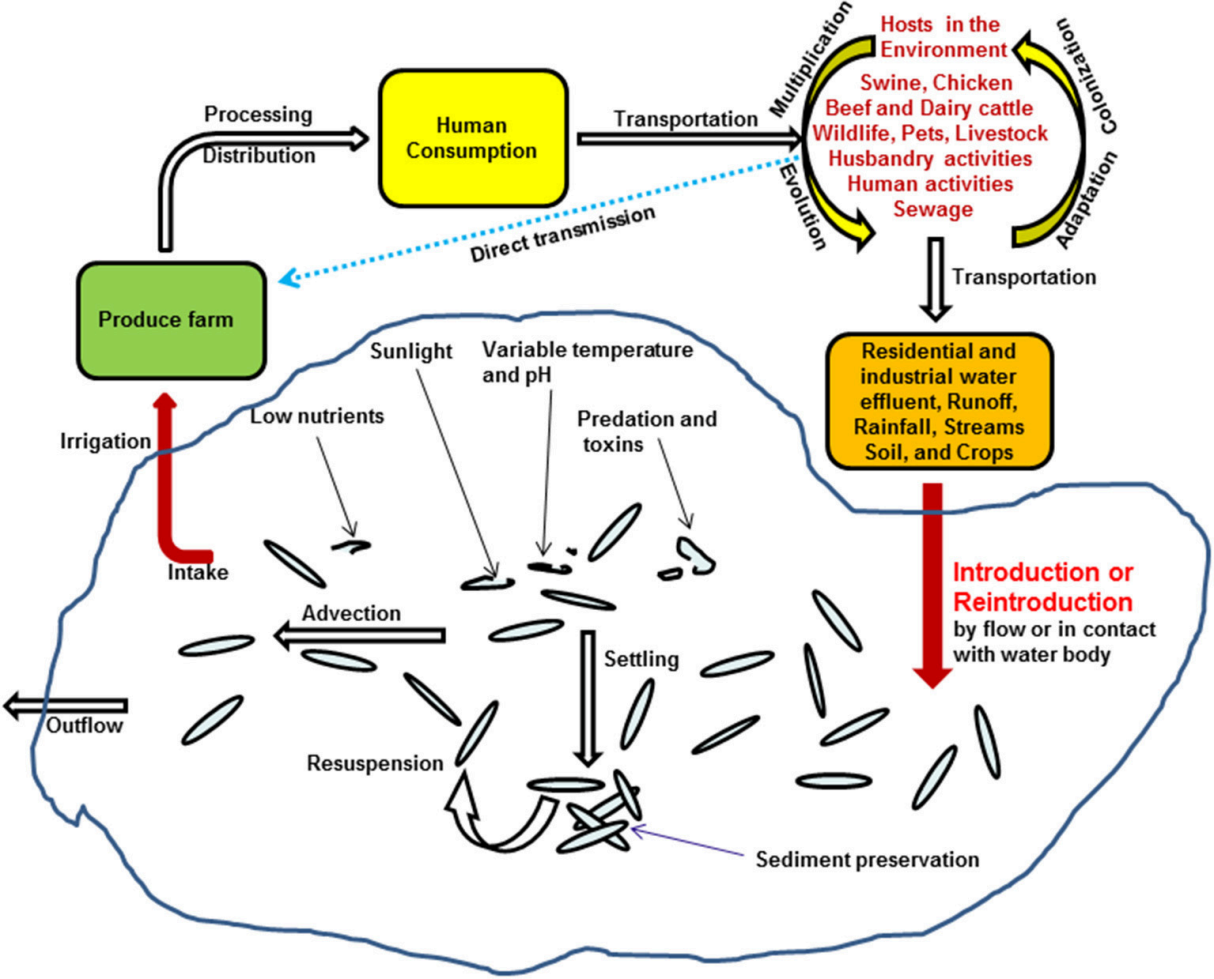
Proposed model of *Salmonella* transmission in irrigation water in the environment, adapted from Li et al. (5).

Besides the elongation of *Salmonella* viability and survivability in water environment, recently, Li et al. also suggested that frequent detection of *Salmonella* in irrigation water might be due to numerous reintroduction events associated with several different hosts in the environment. This was supported by genomic microarray analysis on *Salmonella* Newport isolates (5). On the one hand, produces growing in the field can be directly contaminated by *Salmonella* excreted from proximate environmental hosts, such as swine, chicken, beef, dairy cattle, husbandry or human, other wild lives, or inadequately composited manures applied in the fields (4, 5, 9); on the other hand, these bacteria could also be repeatedly introduced to the irrigation ponds through storm events and rainfalls, survive in water, and reach fresh produce through irrigation, and circulate back to human and other animals (Figure 2) (5).

Furthermore, the prevalence and distribution of *Salmonella* in rivers, streams, or ponds exhibit seasonality, which has been documented in multiple studies (12, 61, 93). Thus, running water formed by rainfall may act as an important vehicle for *Salmonella* transportation. In addition, a number of studies suggest that bacteria can enter a viable but nonculturable (VBNC) state under stress conditions (94), such as low temperature (95), osmotic stress (96), pH changes (97), or nutrient starvation (98). Bacteria in the VBNC state cannot be recovered with routine growth media, but are alive (i.e., they metabolic activity) and these cells can become culturable again on resuscitation (99). *Salmonella* is one of the bacteria reported using VNBC as a survival strategy in harsh environments, including aquatic systems (100–102). The existence of VBNC has been controversial, since although it was reported 30 years ago, there is still little direct conclusive information on the molecular mechanisms underlying VNBC state induction and resuscitation (92, 103).

As mentioned above, many bacterial pathogens are known to survive within free-living protozoa, particularly amoebae (104). In fact, more than 20 species of pathogenic bacteria have been reported to associate with a single species (i.e., *Acanthamoeba*) of amoebae (105). This provides a potential mechanism for bacterial survival in the aquatic environment with the amoebae serving as an environmental reservoir. *Salmonella* Typhimurium was shown to replicate and survive in *Acathamoeba* spp.; however, it was cytotoxic and killed the amoebae (106–108). Douesnard-Malo and Daigle investigated the interactions between *Acathamoeba castellanii* and *Salmonella* Typhi, the etiologic agent of typhoid fever (109). They showed that *S*. Typhi could survive at least 3 weeks when grown in coculture with *A. castellanii* as opposed to less than 10 days when grown as singly cultured bacteria under the same conditions. Additionally, growth rates of amoebae after 14 days were similar in cocultures or when amoebae were singly cultured, suggesting that *S*. Typhi was not cytotoxic to *A. castellanii* (109). These studies suggest that certain species of free-living amoebae can serve as an environmental reservoir for pathogenic *Salmonella* species.

With an increasing number of outbreaks associated with consumption of fresh produce, understanding the mechanisms of produce contamination by foodborne pathogens is useful to develop preventative and processing measures to curtail the microbial populations in produce. A relatively new topic regarding the mechanisms of produce contamination is the concept of internalization of produce by pathogens, which has given rise to debate in the field in the last two decades (43, 110–113). Internalization is defined as the uptake of pathogens through the roots into the intercellular spaces between plant cells and in the plant vasculature tissues, xylem and phloem (114). Internalized pathogens cannot be removed by washing or disinfection and thus can pose a risk for human health if contaminated produce is eaten uncooked (115). The uptake of different foodborne microbes including bacteria (*Salmonella* and *E. coli*) (42, 116–121), viruses (norovirus) (122–124), fungi (125), and protozoa (126) through roots into produce has been reported. However, the presence of pathogens including *Salmonella* inside plant cells remains controversial (35), and further comprehensive and in-depth study is needed on the internalization of produce by pathogens.

## Indirect detection of *Salmonella* in irrigation water

Rapid and accurate detection/estimation of the levels of *Salmonella* and other pathogens is prerequisite for understanding the dynamics of microbial populations and determination of microbial quality of irrigation water (102). The major source of microbial contamination of irrigation water is from fecal origin (4, 127, 128), and thus the microbial quality guidelines of irrigation water are through testing total counts of coliforms, fecal coliforms, *E. coli*, fecal streptococci, and nematode eggs (23, 129). Because total counts of coliforms and fecal coliforms cannot exclude bacteria from nonfecal origin, the presence of *E. coli* is now regarded as a better indicator of microbial quality of irrigation water (23, 129–131). The PSR establishes microbial quality criteria for various uses of agricultural water using generic *E. coli* as an indicator for fecal pollution. However, the use of generic *E. coli* as an index organism for the presence of human pathogens in water sources has been discussed in the literature as well. The PSR also requires that agricultural water must be safe and of adequate sanitary quality for its intended use. It has been shown that the concentration of *E. coli* can predict the level of *Salmonella* present in water (6, 132). However, *Salmonella* could be identified even if *E. coli* counts were below the actionable levels, raising a concern that the satisfaction of the current limit of generic *E. coli* may not necessarily represent the absence of *Salmonella* (6). Accurate estimation/enumeration and isolation of *Salmonella* in water are still indispensable in source-tracking investigations of produce associated outbreaks.

## Direct detection of *Salmonella* in irrigation water

Traditional methods for detection, isolation, and identification of *Salmonella* in water involve nonselective and selective pre-enrichment in liquid culture followed by isolation using selective and differential agar plates. Such methods are laborious and time-consuming, which may take 4–5 days to complete (133). To overcome these limitations, immunoassays such as enzyme-linked immunosorbent assay (ELISA) have been combined with culture methods for detection of *Salmonella* (134), but the application of ELISA is greatly hampered by its poor performance in sensitivity and specificity. Fortunately, a combination of culture-based methods with DNA-based technologies has given a great boost for *Salmonella* detection in various food matrices. DNA-based methods, including conventional and real-time PCR (qPCR), have become the most common methods for the detection of *Salmonella* (135–137), with qPCR being more advantageous due to its specificity, sensitivity, and short turn-round time.

There are two different approaches used in qPCR detection. One approach uses SYBR Green dye to nonspecifically bind to synthesized double DNA (dsDNA). This dye only fluoresces when bound to dsDNA, thus the intensity of fluorescence quantitatively reflects the amount of the newly synthesized dsDNA (138, 139). The other approach is specific, employing a DNA probe with a fluorescent reporter incorporated at one end and a quencher of fluorescence at the other end to prevent detection of fluorescence. Degradation of the probe by 5'−3' nuclease activity of TaqMan polymerase will allow the unquenched emission of fluorescence, and the probe is also complementary to the DNA target and can anneal in each cycle. Thus, the increase of fluorescence can proportionately reflect the amplification of the DNA product. The advantage of this method is that it can be used to detect multiple targets simultaneously with high specificity and sensitivity and is widely used in detection and identification of microorganisms (140–142). The Loop-Mediated Isothermal Amplification (LAMP) can be carried out at a constant temperature and the amplicons are detected by measuring turbidity or fluorescence (143–147). It is a good option for detection of *Salmonella* from waters in rural area or developing countries where thermocycler is not equipped or budget is limited.

Most conventional PCR and qPCR assays for *Salmonella* target the invasive gene (*invA*) because it is an important virulence factor gene (148). This locus is considered to be present in all *Salmonella* spp., including a wide range of *Salmonella* serotypes and absent in other closely related bacterial species and genera (149–151). The biological confirmation of positive PCR results of irrigation water samples with convention methods should proceed, especially when the results are to serve the regulatory purpose. This can be achieved by plating on conventionally-used selective growth agars, including XLD (Xylose Lysine Deoxycholate) (152), HE (Hektoen eteric) (153), and BS (Bismuth sulfite, a modification of Wilson and Blair agar) (154) agar. The presumed *Salmonella* colonies on these plates based on morphology may undergo further biochemical or molecular identification process. *Salmonella* is urease-negative, hence, this trait can also be used for differentiation from some urease-positive bacteria such as *Proteus* and *Citrobacter* species on urea medium with phenol red as in indicator (155).

## Enumeration of *Salmonella* in irrigation water

In general, the level of *Salmonella* cells in food, poultry, produce, or other food matrices is low, and direct enumeration of *Salmonella* cell number has always been a challenge (156). A variety of methods have been established, including direct plating, fluorescence *in situ* hybridization (157), most probable number (MPN) (158), modified MPN methods (132, 156), and qPCR (135, 159, 160). These methods can be applied for enumeration of *Salmonella* populations in irrigation water, however, direct plating sometimes is inefficient due to low level of *Salmonella* cells in water and competitiveness from natural microbiota (161). Direct counting by immunofluorescence-based methods are also not widely used due to low sensitivity in enumeration, problems of antibody quality and linkage of fluorochrome (162). The MPN method is still widely used, in particular, for determination of low concentration of *Salmonella* samples (156), and it has been improved by combination with serology or multiplex PCR in the confirmation step in the MPN method (158, 163). McEgan et al. have developed a modified MPN method for irrigation water. This modified MPN includes a three-by-three MPN dilution test, selective enrichment, plating, biological confirmation, and PCR confirmation, and it normally takes about a week to complete the entire process (132).

Detection of low level of *Salmonella* in food or water samples with molecular methods typically requires a pre-enrichment step to increase the number of the target cells, but the high background microbiota in some foods, such as fresh produce, will also multiply in this process and consequentially complicates the subsequent steps (164). Methods for quantification of *Salmonella* in foods have been developed by using qPCR to detect the DNA of the samples in order to estimate the copy number of a target gene that reflects the quantity of bacterial cells (135, 165). Elimination of the enrichment process presumably may improve the accuracy of the quantification, but this approach is not without drawbacks. First, only a very small amount of DNA template can be added to the PCR reaction mixture, and DNA purity and proportion of target DNA in the total DNA may affect the accuracy of the results. Therefore, it becomes critical to select the appropriate preparation method for separation of bacteria in water or food. The other issue involves the inability of PCR to discriminate DNA from viable cells and dead cells, which may undermine the reliability of the assay. One way to overcome this issue is to detect the presence of RNA (166, 167), which is laborious and can result in false-negative results due to degradation of RNA. The other approach involves using a photoreactive dye propidium monoazide (PMA), which has been incorporated into the PCR to differentiate dead from live bacterial cells in foods (142, 168–170). The principle for PMA's selectivity in detection is based on its ability to penetrate only cell membranes of dead cells, covalently bind to DNA upon light exposure, and subsequently inhibit the modified DNA from amplification by PCR. But caution should be exercised because in some cases inhibition of amplification of PMA-bound DNA from dead cells was found incomplete (171–174).

## Reducing *Salmonella* in irrigation water

Irrigation water is extensively applied during the produce growth stage, and thus the assurance of microbial quality of irrigation water is critical in the protection of produce safety. In the United States, the standards for drinking water (the Safe Drinking Water Act, SDWA) was enacted in 1974, and subsequently the Environmental Protection Agency set the drinking water standards, which requires total coliforms (including the fecal coliforms and *E. coli*) to be 0 per 100 ml water (EPA) (https://www.epa.gov/ground-water-and-drinking-water/national-primary-drinking-water-regulations). Developing general standards for water used for irrigation is rather complicated because the source of water for irrigation can vary greatly with region, season, and climate. Even within the same region, water microbial quality can be quite dynamic. Precipitation, the distance to domesticated animal raising facilities, and the number of wild or domestic animals in the proximity of the water can also constitute substantial changes to the microbial quality of the water (61). However, with the passage of FSMA, and more specifically, the PSR, many growers producing covered crops must now meet certain minimum requirements for the safe use of agricultural water. This may serve as a good solution for improving microbial quality of irrigation water reduction of foodbonre outbreaks assocaited to fresh produces.

## Conclusions

*Salmonella* is frequently detected in surface water, which accounts for nearly half of the water used for irrigation. Trace-back investigations of outbreaks often implicate irrigation water as a source (or a vehicle) for transmission of *Salmonella*. In addition, the bacterium can survive in these aquatic environments by a number of mechanisms, including entry into the VBNC state and/or residing within free-living protozoa. As such, assurance of microbial quality of irrigation water is vital to the mitigation of produce-related foodborne outbreaks.

## Author contributions

BL conceived this project. HL, CW, BL wrote the manuscript. All authors have read and approved the manuscript.

### Conflict of interest statement

The authors declare that the research was conducted in the absence of any commercial or financial relationships that could be construed as a potential conflict of interest. The reviewers LB and AH and handling Editor declared their shared affiliation.

## References

[1] Tauxe R Kruse H Hedberg C Potter M Madden J Wachsmuth K Microbial hazards and emerging issues associated with produce - a preliminary report to the National Advisory Committee on Microbiologic Criteria for Foods. J Food Prot. (1997) 60:1400–8. 10.4315/0362-028X-60.11.1400 31207786

[2] Hanning IB Nutt JD Ricke SC. Salmonellosis outbreaks in the United States due to fresh produce: sources and potential intervention measures. Foodborne Pathog Dis. (2009) 6:635–48. 10.1089/fpd.2008.0232 19580447

[3] Ruby T McLaughlin L Gopinath S Monack D. *Salmonella*'s long-term relationship with its host. FEMS Microbiol Rev. (2012) 36:600–15. 10.1111/j.1574-6976.2012.00332.x 22335190

[4] Andino A Hanning I. *Salmonella* enterica: survival, colonization, and virulence differences among serovars. Scientific World Journal. (2015) 2015:520179. 10.1155/2015/520179 25664339 PMC4310208

[5] Li B Jackson SA Gangiredla J Wang W Liu H Tall BD. Genomic evidence reveals numerous *Salmonella* enterica serovar Newport reintroduction events in Suwannee watershed irrigation ponds. Appl Environ Microbiol. (2015) 81:8243–53. 10.1128/AEM.02179-15 26386063 PMC4644655

[6] Luo ZY Gu GY Ginn A Giurcanu MC Adams P Vellidis G. Distribution and characterization of *Salmonella* enterica isolates from irrigation ponds in the Southeastern United States. Appl Environ Microbiol. (2015) 81:4376–87. 10.1128/AEM.04086-14 25911476 PMC4475880

[7] Whiley H Gardner MG Ross K. A review of *Salmonella* and squamates (lizards, snakes and amphisbians): implications for public health. Pathogens (2017) 6:E38. 10.3390/pathogens6030038 28829352 PMC5617995

[8] Batz MB Hoffman S Morris JG Ranking the Risks: The 10 Pathogen-Food Combinations with the Greatest Burden on Public Health. Gainesville, FL: University of Florida, Emerging Pathogens Institute (2011).

[9] Brandl MT Cox CE Teplitski M. *Salmonella* interactions with plants and their associated microbiota. Phytopathology (2013) 103:316–25. 10.1094/PHYTO-11-12-0295-RVW 23506360

[10] Majowicz SE Musto J Scallan E Angulo FJ Kirk M O'Brien SJ. The global burden of nontyphoidal *Salmonella* gastroenteritis. Clin Infect Dis. (2010) 50:882–9. 10.1086/650733 20158401

[11] Sivapalasingam S Friedman CR Cohen L Tauxe RV. Fresh produce: a growing cause of outbreaks of foodborne illness in the United States, 1973 through 1997. J Food Prot. (2004) 67:2342–53. 10.4315/0362-028X-67.10.2342 15508656

[12] Haley BJ Cole DJ Lipp EK. Distribution, diversity, and seasonality of waterborne *Salmonellae* in a rural watershed. Appl Environ Microbiol. (2009) 75:1248–55. 10.1128/AEM.01648-08 19124594 PMC2648171

[13] Harris L Farber JN Beuchat LR Parish ME Suslow TV Garrett EH Outbreaks associated with fresh produce: incidence, growth, and survival of pathogens in fresh and fresh-cut produce. Comp Rev Food Sci Food Safety (2003) 2:78–141. 10.1111/j.1541-4337.2003.tb00031.x

[14] Walsh C Duffy G Sheridan JJ Fanning S Blair IS McDowell DA Thermal resistance of antibiotic-resistant and antibiotic-sensitive *Salmonella* spp. on chicken meat. J Food Safety (2005) 25:288–302. 10.1111/j.1745-4565.2005.00021.x

[15] Walsh KA Bennett SD Mahovic M Gould LH. Outbreaks associated with cantaloupe, watermelon, and honeydew in the United States, 1973-2011. Foodborne Pathog. Dis. (2014) 11:945–52. 10.1089/fpd.2014.1812 25407556 PMC4627691

[16] Painter JA Hoekstra RM Ayers T Tauxe RV Braden CR Angulo FJ. Attribution of foodborne illnesses, hospitalizations, and deaths to food commodities by using outbreak data, United States, 1998-2008. Emerging Infect Dis. (2013) 19:407–15. 10.3201/eid1903.111866 23622497 PMC3647642

[17] Yang YT Swinburne M. New produce safety regulations: promises and challenges. Public Health Rep. (2016) 131:754–7. 10.1177/0033354916669495 28123220 PMC5230823

[18] Lynch MF Tauxe RV Hedberg CW. The growing burden of foodborne outbreaks due to contaminated fresh produce: risks and opportunities. Epidemiol Infect. (2009) 137:307–15. 10.1017/S0950268808001969 19200406

[19] Denis N Zhang H Leroux A Trudel R Bietlot H Prevalence and trends of bacterial contamination in fresh fruits and vegetables sold at retail in Canada. Food Control (2016) 67:225–34. 10.1016/j.foodcont.2016.02.047

[20] Cook R Tracking Demographics US Fruit Vegetable Consumption Patterns (2011). Available online at: https://arefiles.ucdavis.edu/uploads/filer_public/2014/05/19/blueprintseoeconsumptioncookfinaljan2012figures.pdf

[21] Usda-Ers Economic Research Service, United States Department of Agriculture. (2015). Available online at: https://www.ers.usda.gov/data-products/ag-and-food-statistics-charting-the-essentials/food-availability-and-consumption/

[22] FDA Food Safety Modernization Act: Standards for the Growing, Harvesting, Packing, and Holding of Produce for Human Consumption; Proposed Rule, 2013 (Docket No. FDA-2011-N-0921). Washington, DC: U.S. Food and Drug Administration (2013).

[23] Steele M Odumeru J. Irrigation water as source of foodborne pathogens on fruit and vegetables. J Food Prot. (2004) 67:2839–49. 10.4315/0362-028X-67.12.2839 15633699

[24] Pachepsky Y Shelton DR McLain JET Patel J Mandrell RE Irrigation waters as a source of pathogenic microorganisms in produce: a review. Adv Agron. (2011) 113:73–138. 10.1016/B978-0-12-386473-4.00002-6

[25] Raudales R Parke JL Guy CL Fisher PR Control of waterborne microbes in irrigation:A review. Agric Water Manage. (2014) 143:9–28. 10.1016/j.agwat.2014.06.007

[26] Bihn E Smart CD Hoepting CA Worobo RW Use of surface water in the production of fresh fruits and vegetables: a survey of fresh produce growers and their water management practices. Food Protection Trends (2013) 33:307–14. Available online at: http://www.foodprotection.org/files/food-protection-trends/Sep-Oct-13-bihn.pdf

[27] CPS Agricultural Water: Center for Produce Safety 5 -Year Research Review, Version 1.1. (2014). Available online at: http://www.pma.com/~/media/pma-files/food-safety/cps/cps-research-reportag-water-200813version-11final.pdf

[28] CDC Centers for Disease Control and Prevention. Annual Listing of Foodborne Disease Outbreaks. Atlanta, GA: CDC (2008). Available online at: http://www.cdc.gov/foodborneoutbreaks/outbreak_data.htm

[29] CDC Centers for Disease Control and Prevention: List of Selected Multistate Foodborne Outbreaks and Investigations. (2017). Available online at: https://www.cdc.gov/foodsafety/outbreaks/multistate-outbreaks/outbreaks-list.html

[30] Sivapalasingam S Barrett E Kimura A Van Duyne S De Witt W Ying M. A multistate outbreak of *Salmonella* enterica serotype Newport infection linked to mango consumption: impact of water-dip disinfestation technology. Clin Infect Dis. (2003) 37:1585–90. 10.1086/379710 14689335

[31] Castillo A Mercado I Lucia LM Martinez-Ruiz Y De Leon JP Murano EA. *Salmonella* contamination during production of Cantaloupe: a binational study. J Food Prot. (2004) 67:713–20. 10.4315/0362-028X-67.4.713 15083723

[32] CDC Centers for Disease Control and Prevention. List of Selected Outbreaks. 2006-2015. (2006-2015). Available online at: http://www.cdc.gov/salmonella/outbreaks.html

[33] Fett WF. Inhibition of *Salmonella* enterica by plant-associated pseudomonads *in vitro* and on sprouting alfalfa seed. J Food Prot. (2006) 69:719–28. 10.4315/0362-028X-69.4.719 16629011

[34] Fatica MK Schneider KR. *Salmonella* and produce: survival in the plant environment and implications in food safety. Virulence (2011) 2:573–9. 10.4161/viru.2.6.17880 21971184

[35] Wiedemann A Virlogeux-Payant I Chausse AM Schikora A Velge P. Interactions of *Salmonella* with animals and plants. Front Microbiol. (2015) 5:791. 10.3389/fmicb.2014.00791 25653644 PMC4301013

[36] Leff JW Fierer N. Bacterial communities associated with the surfaces of fresh fruits and vegetables. PLoS ONE (2013) 8:e59310. 10.1371/journal.pone.0059310 23544058 PMC3609859

[37] Goudeau DM Parker CT Zhou Y Sela S Kroupitski Y Brandl MT. The *salmonella* transcriptome in lettuce and cilantro soft rot reveals a niche overlap with the animal host intestine. Appl Environ Microbiol. (2013) 79:250–62. 10.1128/AEM.02290-12 23104408 PMC3536078

[38] Ottesen AR Gonzalez A Bell R Arce C Rideout S Allard M. Co-enriching microflora associated with culture based methods to detect *Salmonella* from tomato phyllosphere. PLoS ONE (2013) 8:e73079. 10.1371/journal.pone.0073079 24039862 PMC3767688

[39] Jarvis KG White JR Grim CJ Ewing L Ottesen AR Beaubrun JJG. Cilantro microbiome before and after nonselective pre-enrichment for *Salmonella* using 16S rRNA and metagenomic sequencing. BMC Microbiol. (2015) 15:160. 10.1186/s12866-015-0497-2 26264042 PMC4534111

[40] Iniguez AL Dong Y Carter HD Ahmer BM Stone JM Triplett EW. Regulation of enteric endophytic bacterial colonization by plant defenses. Mol Plant Microbe Interact. (2005) 18:169–78. 10.1094/MPMI-18-0169 15720086

[41] Schikora A Carreri A Charpentier E Hirt H. The dark side of the salad: *Salmonella* typhimurium overcomes the innate immune response of Arabidopsis thaliana and shows an endopathogenic lifestyle. PLoS ONE (2008) 3:e2279. 10.1371/journal.pone.0002279 18509467 PMC2386236

[42] Zheng J Allard S Reynolds S Millner P Arce G Blodgett RJ. Colonization and internalization of *Salmonella* enterica in tomato plants. Appl Environ Microbiol. (2013) 79:2494–502. 10.1128/AEM.03704-12 23377940 PMC3623171

[43] Guo X Chen JR Brackett RE Beuchat LR. Survival of *Salmonellae* on and in tomato plants from the time of inoculation at flowering and early stages of fruit development through fruit ripening. Appl Environ Microbiol. (2001) 67:4760–4. 10.1128/AEM.67.10.4760-4764.2001 11571182 PMC93229

[44] Stine SW Song I Choi CY Gerba CP. Effect of relative humidity on preharvest survival of bacterial and viral pathogens on the surface of cantaloupe, lettuce, and bell peppers. J Food Prot. (2005) 68:1352–8. 10.4315/0362-028X-68.7.1352 16013370

[45] Barak JD Gorski L Naraghi-Arani P Charkowski AO. *Salmonella* enterica virulence genes are required for bacterial attachment to plant tissue. Appl Environ Microbiol. (2005) 71:5685–91. 10.1128/AEM.71.10.5685-5691.2005 16204476 PMC1265987

[46] Gu G Hu J Cevallos-Cevallos JM Richardson SM Bartz JA Van Bruggen AH. Internal colonization of *Salmonella* enterica serovar Typhimurium in tomato plants. PLoS ONE (2011) 6:e27340. 10.1371/journal.pone.0027340 22096553 PMC3212569

[47] Schikora A Garcia AV Hirt H. Plants as alternative hosts for *Salmonella*. Trends Plant Sci. (2012) 17:245–9. 10.1016/j.tplants.2012.03.007 22513107

[48] Iturriaga MH Tamplin ML Escartin EF. Colonization of tomatoes by *Salmonella* Montevideo is affected by relative and storage temperature. J Food Prot. (2007) 70:30–4. 10.4315/0362-028X-70.1.30 17265856

[49] De Moraes MH Desai P Porwollik S Canals R Perez DR Chu W. *Salmonella* persistence in tomatoes requires a distinct set of metabolic functions identified by transposon insertion sequencing. Appl Environ Microbiol. (2017) 83:e03028-16. 10.1128/AEM.03028-16 28039131 PMC5311394

[50] Barak JD Kramer LC Hao LY. Colonization of tomato plants by *Salmonella* enterica is cultivar dependent, and type 1 trichomes are preferred colonization sites. Appl Environ Microbiol. (2011) 77:498–504. 10.1128/AEM.01661-10 21075871 PMC3020540

[51] Golberg D Kroupitski Y Belausov E Pinto R Sela S. *Salmonella* Typhimurium internalization is variable in leafy vegetables and fresh herbs. Int J Food Microbiol. (2011) 145:250–7. 10.1016/j.ijfoodmicro.2010.12.031 21262550

[52] Kroupitski Y Golberg D Belausov E Pinto R Swartzberg D Granot D. Internalization of *Salmonella* enterica in leaves is induced by light and involves chemotaxis and penetration through open stomata. Appl Environ Microbiol. (2009) 75:6076–86. 10.1128/AEM.01084-09 19648358 PMC2753090

[53] Kroupitski Y Pinto R Brandl MT Belausov E Sela S. Interactions of *Salmonella* enterica with lettuce leaves. J Appl Microbiol. (2009) 106:1876–85. 10.1111/j.1365-2672.2009.04152.x 19239550

[54] Herman KM Hall AJ Gould LH. Outbreaks attributed to fresh leafy vegetables, United States, 1973-2012. Epidemiol Infect. (2015) 143:3011–21. 10.1017/S0950268815000047 25697407 PMC4591532

[55] Parnell TL Harris LJ Suslow TV. Reducing *Salmonella* on cantaloupes and honeydew melons using wash practices applicable to postharvest handling, foodservice, and consumer preparation. Int J Food Microbiol. (2005) 99:59–70. 10.1016/j.ijfoodmicro.2004.07.014 15718029

[56] Boriss H Brunke H Kreith M Commodity Profile: Melons. Davis, CA: University of California Agricultural Issues Center (2006).

[57] Patil R. TJ Ryser E Extent of Listeria monocytogenes transfer during cutting of cantaloupe and honeydew melon. J Food Prot. (2013) 76(Suppl. A):3–99. Available online at: https://iafp.confex.com/iafp/2013/webprogram/Paper4931.html

[58] Jacobsen CS Bech TB Soil survival of *Salmonella* and transfer to freshwater and fresh produce. Food Res Int. (2012) 45:557–66. 10.1016/j.foodres.2011.07.026

[59] Jones LA Worobo RW Smart CD. Plant-pathogenic oomycetes, Escherichia coli strains, and *Salmonella* spp. Frequently found in surface water used for irrigation of fruit and vegetable crops in New York State. Appl Environ Microbiol. (2014) 80:4814–20. 10.1128/AEM.01012-14 24878603 PMC4135776

[60] Jablasone J Brovko LY Griffiths MW A research note: the potential for transfer of *Salmonella* from irrigation water to tomatoes. J Sci Food Agric. (2004) 84:287–9. 10.1002/jsfa.1646

[61] Li B Vellidis G Liu H Jay-Russell M Zhao S Hu Z. Diversity and antimicrobial resistance of *Salmonella* enterica isolates from surface water in Southeastern United States. Appl Environ Microbiol. (2014) 80:6355–65. 10.1128/AEM.02063-14 25107969 PMC4178646

[62] Greene SK Daly ER Talbot EA Demma LJ Holzbauer S Patel NJ. Recurrent multistate outbreak of *Salmonella* Newport associated with tomatoes from contaminated fields, 2005. Epidemiol Infect. (2008) 136:157–65. 10.1017/S095026880700859X 17475091 PMC2870807

[63] Klontz KC Klontz JC Mody RK Hoekstra RM. Analysis of tomato and jalapeno and Serrano pepper imports into the United States from Mexico before and during a National Outbreak of *Salmonella* serotype Saintpaul infections in 2008. J Food Prot. (2010) 73:1967–74. 10.4315/0362-028X-73.11.1967 21219707

[64] Gibbs R Pingault N Mazzucchelli T O'reilly L Mackenzie B Green J. An outbreak of *Salmonella* enterica serotype Litchfield infection in Australia linked to consumption of contaminated papaya. J Food Prot. (2009) 72:1094–8. 10.4315/0362-028X-72.5.1094 19517740

[65] Leifert C Ball K Volakakis N Cooper JM. Control of enteric pathogens in ready-to-eat vegetable crops in organic and ‘low input’ production systems: a HACCP-based approach. J Appl Microbiol. (2008) 105:931–50. 10.1111/j.1365-2672.2008.03794.x 18397255

[66] Santiago P Jimenez-Belenguer A Garcia-Hernandez J Estelles RM Hernandez Perez M Castillo Lopez MA. High prevalence of *Salmonella* spp. in wastewater reused for irrigation assessed by molecular methods. Int J Hyg Environ Health (2018) 221:95–101. 10.1016/j.ijheh.2017.10.007 29107574

[67] Gerba The role of water and water testing in produce safety, In: Fan X Niemira BA Doona CJ Feeherry FE Gravani RB editors. Microbial Safety of Fresh Produce. Oxford, UK: Blackwell-Wiley; IFT Press (2009).

[68] Levantesi C Bonadonna L Briancesco R Grohmann E Toze S Tandoi V *Salmonella* in surface and drinking water: occurrence and water-mediated transmission. Food Res Int. (2012) 45:587–602. 10.1016/j.foodres.2011.06.037

[69] Goss M Richards C. Development of a risk-based index for source water protection planning, which supports the reduction of pathogens from agricultural activity entering water resources. J Environ Manage. (2008) 87:623–32. 10.1016/j.jenvman.2006.12.048 18158213

[70] Bockelmann U Dorries HH Ayuso-Gabella MN De Marcay MS Tandoi V Levantesi C. Quantitative PCR monitoring of antibiotic resistance genes and bacterial pathogens in three european artificial groundwater recharge systems. Appl Environ Microbiol. (2009) 75:154–63. 10.1128/AEM.01649-08 19011075 PMC2612202

[71] Assadian NW Fenn LB Flores-Ortiz MA Ali AS Spatial variability of solutes in a pecan orchard surface-irrigated with untreated effluents in the upper Rio Grande River basin. Agric Water Manage. (1999) 42:143–56. 10.1016/S0378-3774(99)00037-2

[72] Garcia SS Ake C Clement B Huebner HJ Donnelly KC Shalat SL. Initial results of environmental monitoring in the Texas Rio Grande Valley. Environ Int. (2001) 26:465–74. 10.1016/S0160-4120(01)00027-7 11485214

[73] Baudart J Lemarchand K Brisabois A Lebaron P. Diversity of *Salmonella* strains isolated from the aquatic environment as determined by serotyping and amplification of the ribosomal DNA spacer regions. Appl Environ Microbiol. (2000) 66:1544–52. 10.1128/AEM.66.4.1544-1552.2000 10742240 PMC92021

[74] Baudart J Grabulos J Barusseau JP Lebaron P *Salmonella* spp. and fecal coliform loads in coastal waters from a point vs. nonpoint source of pollution. J Environ Qual. (2000) 29:241–50. 10.2134/jeq2000.00472425002900010031x

[75] Su LH Chiu CH Chu C Ou JT. Antimicrobial resistance in nontyphoid *Salmonella* serotypes: a global challenge. Clin Infect Dis. (2004) 39:546–51. 10.1086/422726 15356819

[76] Molbak K. Human health consequences of antimicrobial drug-resistant *Salmonella* and other foodborne pathogens. Clin Infect Dis. (2005) 41:1613–20. 10.1086/497599 16267734

[77] Odjadjare EC Olaniran AO. Prevalence of antimicrobial resistant and virulent *Salmonella* spp. in treated effluent and receiving aquatic milieu of wastewater treatment plants in Durban, South Africa. Int J Environ Res Public Health (2015) 12:9692–713. 10.3390/ijerph120809692 26295245 PMC4555307

[78] Islam M Morgan J Doyle MP Phatak SC Millner P Jiang XP Fate of *Salmonella* enterica serovar Typhimurium on carrots and radishes grown in fields treated with contaminated manure composts or irrigation water. Appl Environ Microbiol. (2004) 70:2497–502. 10.1128/AEM.70.4.2497-2502.2004 15066849 PMC383101

[79] Kay D Crowther J Stapleton CM Wyer MD Fewtrell L Edwards A. Faecal indicator organism concentrations in sewage and treated effluents. Water Res. (2008) 42:442–54. 10.1016/j.watres.2007.07.036 17709126

[80] Rose JB Huffman DE Riley K Farrah SR Lukasik JO Hamann CL. Reduction of enteric microorganisms at the Upper Occoquan Sewage Authority Water Reclamation Plant. Water Environ Res. (2001) 73:711–20. 10.2175/106143001X143457 11833765

[81] Howard I Espigares E Lardelli P Martin JL Espigares M. Evaluation of microbiological and physicochemical indicators for wastewater treatment. Environ Toxicol. (2004) 19:241–9. 10.1002/tox.20016 15101039

[82] Naidoo S Olaniran AO. Treated wastewater effluent as a source of microbial pollution of surface water resources. Int J Environ Res Public Health (2014) 11:249–70. 10.3390/ijerph110100249 24366046 PMC3924443

[83] Woodward DL Khakhria R Johnson WM. Human salmonellosis associated with exotic pets. J Clin Microbiol. (1997) 35:2786–790. 9350734 10.1128/jcm.35.11.2786-2790.1997 PMC230062

[84] Sha Q Forstner MR Hahn D. Diversity of *Salmonella* in biofilms and water in a headwater ecosystem. FEMS Microbiol Ecol. (2013) 83:642–9. 10.1111/1574-6941.12021 23025800

[85] Liao CH Shollenberger LM. Survivability and long-term preservation of bacteria in water and in phosphate-buffered saline. Lett Appl Microbiol. (2003) 37:45–50. 10.1046/j.1472-765X.2003.01345.x 12803555

[86] Wilkes G Edge TA Gannon VPJ Jokinen C Lyautey E Neumann NF. Associations among pathogenic bacteria, parasites, and environmental and land use factors in multiple mixed-use watersheds. Water Res. (2011) 45:5807–25. 10.1016/j.watres.2011.06.021 21889781

[87] Wanjugi P Harwood VJ. The influence of predation and competition on the survival of commensal and pathogenic fecal bacteria in aquatic habitats. Environ Microbiol. (2013) 15:517–26. 10.1111/j.1462-2920.2012.02877.x 23013262

[88] Gaertner JP Mendoza JA Forstner MRJ Hahn D. Recovery of *Salmonella* from biofilms in a headwater spring ecosystem. J Water Health (2011) 9:458–66. 10.2166/wh.2011.173 21976193

[89] Sha Q Gunathilake A Forstner MR Hahn D. Temporal analyses of the distribution and diversity of *Salmonella* in natural biofilms. Syst Appl Microbiol. (2011) 34:353–9. 10.1016/j.syapm.2011.01.005 21536398

[90] Hendricks CW. Increased recovery rate of *Salmonellae* from stream bottom sediments versus surface waters. Appl Microbiol. (1971) 21:379–80. 5544303 10.1128/am.21.2.379-380.1971 PMC377184

[91] Morinigo MA Borrego JJ Romero P. Comparative study of different methods for detection and enumeration of *Salmonella* spp. in natural waters. J Appl Bacteriol. (1986) 61:169–76. 10.1111/j.1365-2672.1986.tb04272.x 3771414

[92] Moore BC Martinez E Gay JM Rice DH. Survival of *Salmonella* enterica in freshwater and sediments and transmission by the aquatic midge Chironomus tentans (Chironomidae: Diptera). Appl Environ Microbiol. (2003) 69:4556–60. 10.1128/AEM.69.8.4556-4560.2003 12902242 PMC169145

[93] Huang KH Hsu BM Chou MY Tsai HL Kao PM Wang HJ. Application of molecular biological techniques to analyze *Salmonella* seasonal distribution in stream water. FEMS Microbiol Lett. (2014) 352:87–96. 10.1111/1574-6968.12381 24417320

[94] Xu HS Roberts N Singleton FL Attwell RW Grimes DJ Colwell RR Survival and viability of nonculturable escherichia-coli and vibrio-cholerae in the estuarine and marine-environment. Microb Ecol. (1982) 8:313–23. 10.1007/BF02010671 24226049

[95] Cook KL Bolster CH. Survival of Campylobacter jejuni and Escherichia coli in groundwater during prolonged starvation at low temperatures. J Appl Microbiol. (2007) 103:573–83. 10.1111/j.1365-2672.2006.03285.x 17714390

[96] Kusumoto A Asakura H Kawamoto K. General stress sigma factor RpoS influences time required to enter the viable but non-culturable state in *Salmonella* enterica. Microbiol Immunol. (2012) 56:228–37. 10.1111/j.1348-0421.2012.00428.x 22256797

[97] Tholozan JL Cappelier JM Tissier JP Delattre G Federighi M. Physiological characterization of viable-but-nonculturable Campylobacter jejuni cells. Appl Environ Microbiol. (1999) 65:1110–6. 10049870 10.1128/aem.65.3.1110-1116.1999 PMC91151

[98] Gupte AR De Rezende CLE Joseph SW. Induction and resuscitation of viable but nonculturable *Salmonella* enterica serovar typhimurium DT104. Appl Environ Microbiol. (2003) 69:6669–75. 10.1128/AEM.69.11.6669-6675.2003 14602627 PMC262293

[99] Oliver JD. The viable but nonculturable state in bacteria. J Microbiol. (2005) 43:93–100. Available online at: https://pdfs.semanticscholar.org/e661/934dca6bb0dbb31a8781f3193232b7b5a8a4.pdf 15765062

[100] Roszak DB Grimes DJ Colwell RR. Viable but nonrecoverable stage of *Salmonella*-enteritidis in aquatic systems. Can J Microbiol. (1984) 30:334–8. 10.1139/m84-049 6372975

[101] Desmonts C Minet J Colwell R Cormier M Fluorescent-antibody method useful for detecting viable but nonculturable *Salmonella* spp. in chlorinated waste-water. Appl Environ Microbiol. (1990) 56:1448–52. 2187414 10.1128/aem.56.5.1448-1452.1990 PMC184427

[102] Morishige Y Koike A Tamura-Ueyama A Amano F. Induction of viable but nonculturable *Salmonella* in exponentially grown cells by exposure to a low-humidity environment and their resuscitation by catalase. J Food Prot. (2017) 80:288–94. 10.4315/0362-028X.JFP-16-183 28221986

[103] Pinto D Santos MA Chambel L. Thirty years of viable but nonculturable state research: unsolved molecular mechanisms. Crit Rev Microbiol. (2015) 41:61–76. 10.3109/1040841X.2013.794127 23848175

[104] Molmeret M Horn M Wagner M Santic M Abu Kwaik Y. Amoebae as training grounds for intracellular bacterial pathogens. Appl Environ Microbiol. (2005) 71:20–8. 10.1128/AEM.71.1.20-28.2005 15640165 PMC544274

[105] Winiecka-Krusnell J Linder E. Bacterial infections of free-living amoebae. Res Microbiol. (2001) 152:613–9. 10.1016/S0923-2508(01)01240-2 11605981

[106] Gaze WH Burroughs N Gallagher MP Wellington EMH. Interactions between *Salmonella* typhimurium and Acanthamoeba polyphaga, and observation of a new mode of intracellular growth within contractile vacuoles. Microb Ecol. (2003) 46:358–69. 10.1007/s00248-003-1001-3 14502413

[107] Tezcan-Merdol D Ljungstrom M Winiecka-Krusnell J Linder E Engstrand L Rhen M. Uptake and replication of *Salmonella* enterica in Acanthamoeba rhysodes. Appl Environ Microbiol. (2004) 70:3706–14. 10.1128/AEM.70.6.3706-3714.2004 15184177 PMC427739

[108] Feng Y Hsiao YH Chen HL Chu CS Tang P Chiu CH. Apoptosis-like cell death induced by *Salmonella* in Acanthamoeba rhysodes. Genomics (2009) 94:132–7. 10.1016/j.ygeno.2009.05.004 19446019

[109] Douesnard-Malo F Daigle F. Increased persistence of *Salmonella* enterica serovar Typhi in the presence of Acanthamoeba castellanii. Appl Environ Microbiol. (2011) 77:7640–6. 10.1128/AEM.00699-11 21926221 PMC3209172

[110] Guo X Chen JR Brackett RE Beuchat LR. Survival of *Salmonella* on tomatoes stored at high relative humidity, in soil, and on tomatoes in contact with soil. J Food Prot. (2002) 65:274–9. 10.4315/0362-028X-65.2.274 11848557

[111] Kutter S Hartmann A Schmid M. Colonization of barley (Hordeum vulgare) with *Salmonella* enterica and *Listeria* spp. FEMS Microbiol Ecol. (2006) 56:262–71. 10.1111/j.1574-6941.2005.00053.x 16629755

[112] Franz E Visser AA Van Diepeningen AD Klerks MM Termorshuizen AJ Van Bruggen AH. Quantification of contamination of lettuce by GFP-expressing *Escherichia coli* O157:H7 and *Salmonella* enterica serovar Typhimurium. Food Microbiol. (2007) 24:106–12. 10.1016/j.fm.2006.03.002 16943102

[113] Sharma M Ingram DT Patel JR Millner PD Wang X Hull AE. A novel approach to investigate the uptake and internalization of Escherichia coli O157:H7 in spinach cultivated in soil and hydroponic medium. J Food Prot. (2009) 72:1513–20. 10.4315/0362-028X-72.7.1513 19681280

[114] Hirneisen KA Sharma M Kniel KE. Human enteric pathogen internalization by root uptake into food crops. Foodborne Pathog Dis. (2012) 9:396–405. 10.1089/fpd.2011.1044 22458717

[115] Fornefeld E Schierstaedt J Jechalke S Grosch R Schikora A Smalla K. Persistence of *Salmonella* Typhimurium LT2 in soil enhanced after growth in lettuce medium. Front Microbiol. (2017) 8:757. 10.3389/fmicb.2017.00757 28503171 PMC5408095

[116] Bernstein N Sela S Neder-Lavon S. Assessment of contamination potential of lettuce by *Salmonella* enterica serovar Newport added to the plant growing medium. J Food Prot. (2007) 70:1717–22. 10.4315/0362-028X-70.7.1717 17685348

[117] Bernstein N Sela S Pinto R Ioffe M. Evidence for internalization of Escherichia coli into the aerial parts of maize via the root system. J Food Prot. (2007) 70:471–5. 10.4315/0362-028X-70.2.471 17340885

[118] Brandl MT Amundson R. Leaf age as a risk factor in contamination of lettuce with Escherichia coli O157:H7 and *Salmonella* enterica. Appl Environ Microbiol. (2008) 74:2298–306. 10.1128/AEM.02459-07 18310433 PMC2293143

[119] Lopez-Velasco G Sbodio A Tomas-Callejas A Wei P Tan KH Suslow TV. Assessment of root uptake and systemic vine-transport of *Salmonella* enterica sv. Typhimurium by melon (Cucumis melo) during field production. Int J Food Microbiol. (2012) 158:65–72. 10.1016/j.ijfoodmicro.2012.07.005 22824339

[120] Gomez-Lopez VM Marin A Allende A Beuchat LR Gil MI. Postharvest handling conditions affect internalization of *Salmonella* in baby spinach during washing. J Food Prot. (2013) 76:1145–51. 10.4315/0362-028X.JFP-12-539 23834788

[121] Zhang Y Sallach JB Hodges L Snow DD Bartelt-Hunt SL Eskridge KM. Effects of soil texture and drought stress on the uptake of antibiotics and the internalization of *Salmonella* in lettuce following wastewater irrigation. Environ Pollut. (2016) 208:523–31. 10.1016/j.envpol.2015.10.025 26552531

[122] Wei J Jin Y Sims T Kniel KE. Manure- and biosolids-resident murine norovirus 1 attachment to and internalization by Romaine lettuce. Appl Environ Microbiol. (2010) 76:578–83. 10.1128/AEM.02088-09 19933344 PMC2805210

[123] Wang Q Markland S Kniel KE. Inactivation of human norovirus and its surrogates on alfalfa seeds by aqueous ozone. J Food Prot. (2015) 78:1586–91. 10.4315/0362-028X.JFP-15-029 26219375

[124] Wang Q Kniel KE. Survival and transfer of murine norovirus within a hydroponic system during kale and mustard microgreen harvesting. Appl Environ Microbiol. (2016) 82:705–13. 10.1128/AEM.02990-15 26567309 PMC4711135

[125] Vorholt JA. Microbial life in the phyllosphere. Nat Rev Microbiol. (2012) 10:828–40. 10.1038/nrmicro2910 23154261

[126] Deng W Gibson KE. Interaction of microorganisms within leafy green phyllospheres: where do human noroviruses fit in? Int J Food Microbiol. (2017) 258:28–37. 10.1016/j.ijfoodmicro.2017.07.010 28755583

[127] Yan T Sadowsky MJ. Determining sources of fecal bacteria in waterways. Environ Monit Assess. (2007) 129:97–106. 10.1007/s10661-006-9426-z 17072547

[128] Aijuka M Charimba G Hugo CJ Buys EM. Characterization of bacterial pathogens in rural and urban irrigation water. J Water Health (2015) 13:103–17. 10.2166/wh.2014.228 25719470

[129] Edberg SC Rice EW Karlin RJ Allen MJ *Escherichia coli*: the best biological drinking water indicator for public health protection. Symp Ser Soc Appl Microbiol. (2000) 88:106S–16S. 10.1111/j.1365-2672.2000.tb05338.x 10880185

[130] Campbell LM Michaels G Klein RD Roth IL. Isolation of Klebsiella pneumoniae from lake water. Can. J Microbiol. (1976) 22:1762–7. 10.1139/m76-260 795534

[131] Barrell RA Hunter PR Nichols G. Microbiological standards for water and their relationship to health risk. Commun Dis Public Health (2000) 3:8–13. Available online at: http://martechnicltd.com/sales/docs/LGL_Microbiological_Standards_forWater_HPA.pdf 10743312

[132] McEgan R Mootian G Goodridge LD Schaffner DW Danyluk MD. Predicting *Salmonella* populations from biological, chemical, and physical indicators in Florida surface waters. Appl Environ Microbiol. (2013) 79:4094–105. 10.1128/AEM.00777-13 23624476 PMC3697547

[133] Bell RL Jarvis KG Ottesen AR McFarland MA Brown EW. Recent and emerging innovations in *Salmonella* detection: a food and environmental perspective. Microb Biotechnol. (2016) 9:279–92. 10.1111/1751-7915.12359 27041363 PMC4835567

[134] Mansfield LP Forsythe SJ. The detection of *Salmonella* using a combined immunomagnetic separation and ELISA end-detection procedure. Lett Appl Microbiol. (2000) 31:279–83. 10.1046/j.1472-765x.2000.00811.x 11068907

[135] Wolffs PFG Glencross K Thibaudeau R Griffiths MW. Direct quantitation and detection of *Salmonellae* in biological samples without enrichment, using two-step filtration and real-time PCR. Appl Environ Microbiol. (2006) 72:3896–900. 10.1128/AEM.02112-05 16751494 PMC1489624

[136] Eriksson E Aspan A. Comparison of culture, ELISA and PCR techniques for *Salmonella* detection in faecal samples for cattle, pig and poultry. BMC Vet Res. (2007) 3:21. 10.1186/1746-6148-3-21 17888169 PMC2110889

[137] Malorny B Lofstrom C Wagner M Kramer N Hoorfar J. Enumeration of *Salmonella* bacteria in food and feed samples by real-time PCR for quantitative microbial risk assessment. Appl Environ Microbiol. (2008) 74:1299–304. 10.1128/AEM.02489-07 18165357 PMC2258648

[138] Fukushima H Tsunomori Y Seki R. Duplex real-time SYBR green PCR assays for detection of 17 species of food- or waterborne pathogens in stools. J Clin Microbiol. (2003) 41:5134–46. 10.1128/JCM.41.11.5134-5146.2003 14605150 PMC262470

[139] Barbau-Piednoir E Bertrand S Mahillon J Roosens NH Botteldoorn N SYBRA (R) Green qPCR *Salmonella* detection system allowing discrimination at the genus, species and subspecies levels. Appl Microbiol Biotechnol. (2013) 97:9811–24. 10.1007/s00253-013-5234-x 24113820 PMC3825158

[140] Hoorfar J Ahrens P Radstrom P. Automated 5′ nuclease PCR assay for identification of *Salmonella enterica*. J Clin Microbiol. (2000) 38:3429–35. Available online at: http://jcm.asm.org/content/38/9/3429.full.pdf+html 10970396 10.1128/jcm.38.9.3429-3435.2000 PMC87399

[141] Malorny B Paccassoni E Fach P Bunge C Martin A Helmuth R. Diagnostic real-time PCR for detection of *Salmonella* in food. Appl Environ Microbiol. (2004) 70:7046–52. 10.1128/AEM.70.12.7046-7052.2004 15574899 PMC535175

[142] Li B Chen JQ. Real-time PCR methodology for selective detection of viable Escherichia coli O157:H7 cells by targeting Z3276 as a genetic marker. Appl Environ Microbiol. (2012) 78:5297–304. 10.1128/AEM.00794-12 22635992 PMC3416439

[143] Mori Y Nagamine K Tomita N Notomi T. Detection of loop-mediated isothermal amplification reaction by turbidity derived from magnesium pyrophosphate formation. Biochem Biophys Res Commun. (2001) 289:150–4. 10.1006/bbrc.2001.5921 11708792

[144] Hara-Kudo Y Yoshino M Kojima T Ikedo M. Loop-mediated isothermal amplification for the rapid detection of *Salmonella*. FEMS Microbiol Lett. (2005) 253:155–61. 10.1016/j.femsle.2005.09.032 16242860

[145] Tomita N Mori Y Kanda H Notomi T. Loop-mediated isothermal amplification (LAMP) of gene sequences and simple visual detection of products. Nat Protoc. (2008) 3:877–82. 10.1038/nprot.2008.57 18451795

[146] Chen S Wang F Beaulieu JC Stein RE Ge B. Rapid detection of viable *Salmonellae* in produce by coupling propidium monoazide with loop-mediated isothermal amplification. Appl Environ Microbiol. (2011) 77:4008–16. 10.1128/AEM.00354-11 21498750 PMC3131628

[147] Oscorbin IP Belousova EA Zakabunin AI Boyarskikh UA Filipenko ML. Comparison of fluorescent intercalating dyes for quantitative loop-mediated isothermal amplification (qLAMP). BioTechniques (2016) 61:20–5. 10.2144/000114432 27401670

[148] Galan JE Ginocchio C Costeas P. Molecular and functional characterization of the *Salmonella* invasion gene invA: homology of InvA to members of a new protein family. J Bacteriol. (1992) 174:4338–49. 10.1128/jb.174.13.4338-4349.1992 1624429 PMC206218

[149] Rahn K De Grandis SA Clarke RC McEwen SA Galan JE Ginocchio C. Amplification of an invA gene sequence of *Salmonella* typhimurium by polymerase chain reaction as a specific method of detection of *Salmonella*. Mol Cell Probes (1992) 6:271–9. 10.1016/0890-8508(92)90002-F 1528198

[150] Malorny B Hoorfar J Bunge C Helmuth R. Multicenter validation of the analytical accuracy of *Salmonella* PCR: towards an international standard. Appl Environ Microbiol. (2003) 69:290–6. 10.1128/AEM.69.1.290-296.2003 12514007 PMC152403

[151] Mainar-Jaime RC Andres S Vico JP San Roman B Garrido V Grillo MJ. Sensitivity of the ISO 6579:2002/Amd 1:2007 standard method for detection of *Salmonella* spp. on mesenteric lymph nodes from slaughter pigs. J Clin Microbiol. (2013) 51:89–94. 10.1128/JCM.02099-12 23100334 PMC3536256

[152] Zajc-Satler J Gragas AZ. Xylose lysine deoxycholate agar for the isolation of *Salmonella* and Shigella from clinical specimens. Zentralbl Bakteriol Orig. A (1977) 237:196–200. 848209

[153] Goo VY Ching GQ Gooch JM. Comparison of brilliant green agar and Hektoen enteric agar media in the isolation of *Salmonellae* from food products. Appl Microbiol. (1973) 26:288–92. 4584576 10.1128/am.26.3.288-292.1973 PMC379776

[154] Wilson WJ Blair EM. Use of a glucose bismuth sulphite iron medium for the isolation of *B. typhosus* and *B. proteus*. J Hyg (Lond.) (1927) 26:374–91. 10.1017/S0022172400009220 20474937 PMC2167806

[155] Christensen WB. Urea Decomposition as a Means of Differentiating Proteus and Paracolon Cultures from Each Other and from *Salmonella* and Shigella Types. J. Bacteriol. (1946) 52:461–6. 16561200 10.1128/jb.52.4.461-466.1946 PMC518212

[156] Fravalo P Hascoet Y Le Fellic M Queguiner S Petton J Salvat G Convenient method for rapid and quantitative assessment of *Salmonella* enterica contamination: the mini-MSRV MPN technique. J Rapid Methods Autom Microbiol. (2003) 11:81–8. 10.1111/j.1745-4581.2003.tb00031.x

[157] Stender H Oliveira K Rigby S Bargoot F Coull J. Rapid detection, identification, and enumeration of Escherichia coli by fluorescence *in situ* hybridization using an array scanner. J Microbiol Methods (2001) 45:31–9. 10.1016/S0167-7012(01)00218-4 11295195

[158] Humbert FS Salvat G Lalande F Colin P Miniaturized most probable number and enrichment serology technique for the enumeration of *Salmonella* spp. on poultry carcasses. J Food Protect. (1997) 60:1306–11. 10.4315/0362-028X-60.11.1306 31207776

[159] Malorny B Tassios PT Radstrom P Cook N Wagner M Hoorfar J. Standardization of diagnostic PCR for the detection of foodborne pathogens. Int J Food Microbiol. (2003) 83:39–48. 10.1016/S0168-1605(02)00322-7 12672591

[160] Mackay IM. Real-time PCR in the microbiology laboratory. Clin Microbiol Infect. (2004) 10:190–212. 10.1111/j.1198-743X.2004.00722.x 15008940

[161] Nye KJ Fallon D Frodsham D Gee B Graham C Howe S. An evaluation of the performance of XLD, DCA, MLCB, and ABC agars as direct plating media for the isolation of *Salmonella* enterica from faeces. J Clin Pathol. (2002) 55:286–8. 10.1136/jcp.55.4.286 11919214 PMC1769632

[162] Wang XL Slavik MF. Rapid detection of *Salmonella* in chicken washes by immunomagnetic separation and flow cytometry. J Food Prot. (1999) 62:717–23. 10.4315/0362-028X-62.7.717 10419261

[163] Soumet C Ermel G Rose V Rose N Drouin P Salvat G. Identification by a multiplex PCR-based assay of *Salmonella* typhimurium and *Salmonella* Enteritidis strains from environmental swabs of poultry houses. Lett Appl Microbiol. (1999) 29:1–6. 10.1046/j.1365-2672.1999.00559.x 10432625

[164] Malorny B Junker E Helmuth R. Multi-locus variable-number tandem repeat analysis for outbreak studies of *Salmonella* enterica serotype Enteritidis. BMC Microbiol. (2008) 8:84. 10.1186/1471-2180-8-84 18513386 PMC2430564

[165] Guy RA Kapoor A Holicka J Shepherd D Horgen PA. A rapid molecular-based assay for direct quantification of viable bacteria in slaughterhouses. J. Food Prot. (2006) 69:1265–72. 10.4315/0362-028X-69.6.1265 16786844

[166] Miller ND Draughon FA D'souza DH. Real-time reverse-transcriptase-polymerase chain reaction for *Salmonella* enterica detection from jalapeno and serrano peppers. Foodborne Pathog Dis. (2010) 7:367–73. 10.1089/fpd.2009.0398 19911882

[167] Techathuvanan C Draughon FA D'souza DH. Real-time reverse transcriptase PCR for the rapid and sensitive detection of *Salmonella* typhimurium from pork. J Food Prot. (2010) 73:507–514. 10.4315/0362-028X-73.3.507 20202337

[168] Bohaychuk VM Gensler GE McFall ME King RK Renter DG. A real-time PCR assay for the detection of *Salmonella* in a wide variety of food and food-animal matricest. J Food Prot. (2007) 70:1080–7. 10.4315/0362-028X-70.5.1080 17536664

[169] Nocker A Sossa KE Camper AK. Molecular monitoring of disinfection efficacy using propidium monoazide in combination with quantitative PCR. J Microbiol Methods (2007) 70:252–260. 10.1016/j.mimet.2007.04.014 17544161

[170] Li BG Chen JQ. Development of a sensitive and specific qPCR assay in conjunction with propidium monoazide for enhanced detection of live *Salmonella* spp. in food. BMC Microbiol. (2013) 13:273. 10.1186/1471-2180-13-273 24289661 PMC4219372

[171] Luo JF Lin WT Guo Y. Method to detect only viable cells in microbial ecology. Appl Microbiol Biotechnol. (2010) 86:377–84. 10.1007/s00253-009-2373-1 20024544

[172] Contreras PJ Urrutia H Sossa K Nocker A. Effect of PCR amplicon length on suppressing signals from membrane-compromised cells by propidium monoazide treatment. J Microbiol Methods (2011) 87:89–95. 10.1016/j.mimet.2011.07.016 21821068

[173] Soejima T Schlitt-Dittrich F Yoshida S. Rapid detection of viable bacteria by nested polymerase chain reaction via long DNA amplification after ethidium monoazide treatment. Anal Biochem. (2011) 418:286–94. 10.1016/j.ab.2011.06.033 21771580

[174] Schnetzinger F Pan Y Nocker A. Use of propidium monoazide and increased amplicon length reduce false-positive signals in quantitative PCR for bioburden analysis. Appl Microbiol Biotechnol. (2013) 97:2153–62. 10.1007/s00253-013-4711-6 23354451

